# Toll-like receptors (TLRs) and mannan-binding lectin (MBL): On constant alert in a hostile environment

**DOI:** 10.3109/03009734.2010.545494

**Published:** 2011-04-12

**Authors:** Ingrid-Maria Bergman

**Affiliations:** Linnaeus University, School of Natural Sciences, Kalmar, Sweden

**Keywords:** Innate immunity, MBL, molecular biology, positive selection, recognition, TLRs

## Abstract

In the beginning were neither B cells nor T cells nor antibodies, but innate immune defense alone. The primary functional theme of innate immunity is the distinction between self and non-self, which is maintained by a vast number of cellular and subcellular components. In this context, the immense importance of the Toll-like receptors (TLRs) is well established. Positive (Darwinian) selection seems to be acting on the ligand-binding domains of these molecules, suggesting a selection pattern similar to that previously observed in the MHC proteins. In sharp contrast to TLRs, the biological significance of mannan-binding lectin (MBL) is controversial, and, concerning humans, it has been suggested that low concentration of MBL in serum represents a selective advantage. In this mini-review, based on a doctoral thesis, evolutionary aspects of TLRs and MBL are discussed.

## Innate immunity—a prerequisite for survival

The vertebrate immune defense has two arms: the innate and the adaptive immune systems. These are ideal partners because of their different recognition strategies, which have complementary strengths and weaknesses (1). Through the use of pattern recognition receptors (PRRs), the innate immune defense is highly efficient at distinguishing self from non-self, but has—due to its non-clonal nature and despite safety measures like complement control proteins (2) and the activities of sialic acid-specific lectins (siglecs) (3) and signaling regulatory protein α (SIRPα) (4)—the potential to cause significant collateral damage to self tissue. The adaptive immune defense, relying on clonally expressed receptors, is highly effective in targeting the immune response against infection while sparing uninfected tissue; however, the cells of the adaptive immune defense cannot by themselves reliably determine the origin of the antigen they are specific for. By joined forces, the evolutionary ancient and immediate innate immune defense and the younger, highly specific, but temporally delayed adaptive immune defense maximize the survival potential of their host (1).

In bony fish—the most diverse group of vertebrates (5)—several innate immunity components are more active and diverse than their mammalian counterparts (6). Among piscine Toll-like receptors (TLRs), there are examples of duplicated genes and wider ligand repertoires compared to the mammalian equivalents (7). Two mannan-binding lectins (MBLs) have been detected in rainbow trout, but there are indications that these may be members of a larger trout MBL family (8). Certain complement factors are more diverse in fish than in mammals (6), and the molecules of the alternative pathway of complement display five to ten times higher titers in fish (9). These differences between mammalian and piscine innate immunity may represent a compensatory strategy, since the adaptive immune response in fish is limited and slow. Also, the adaptive immune defense generally develops late in marine species, making the innate immune defense their only protection against pathogens during the first two or three months after hatching. The lower anti-pathogen activity of the innate immune defense in mammals can be interpreted as an evolutionary shift in function, in which communication with the adaptive immune defense and maintenance of homeostasis is becoming increasingly important (6).

The innate immune defense has previously been considered unsophisticated and non-specific, a ‘little sister’ of the more important adaptive immune defense. However, the growing awareness of the immense importance of TLRs has promoted innate immunity research and led to new insights concerning its elaborate nature and specificity, stressing its role as the primary defense system in all species, including those which possess an adaptive immune defense. In mammals, TLRs and MBL occupy central positions at the border between innate and adaptive immunity: complement, initiated by MBL, co-ordinates adaptive immune functions (10), and dendritic cells, the most specialized antigen-presenting cells, are known to express TLRs (11). This mini-review, based on a doctoral thesis, aims to describe evolutionary aspects of TLRs and MBL and contribute to the discussion on how natural selection is shaping these molecules for their roles in host defense.

## Who's there? The recognition strategies of the innate immune defense

**Figure 1.**
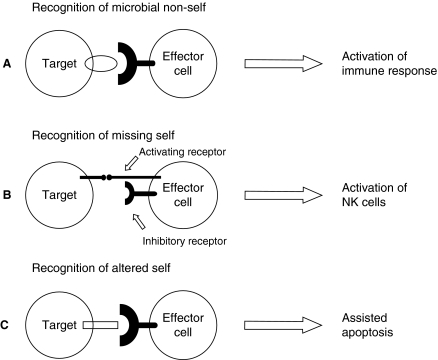
Three recognition strategies used by the innate immune defense. A: Recognition of microbial non-self induces immune response. B: Natural killer cells interact with target cells through activating and inhibitory receptors. When both types of receptors are engaged, the inhibitory receptors are dominant and the natural killer cell is not activated. However, if self marker molecules are missing, the natural killer cell is released from its state of inhibition. C: Expression of markers of altered self flags the cell for destruction.

The main task of the innate immune defense is to discriminate between self and non-self. This is achieved through three strategies of recognition: recognition of microbial non-self, recognition of missing self, and recognition of altered self (Figure 1) (12).

Recognition of microbial non-self is based on the interaction between PRRs—such as TLRs and MBL—and micro-organism-associated molecular patterns (MAMPs). MAMPs are conserved structures, invariant in a particular class of micro-organisms, which are not present within the host. Furthermore, these structures are essential for the viability/adaptive fitness of the micro-organism and thus not easily discarded (13). Secreted PRRs, like MBL, bind to microbial cells and flag them for phagocytosis or elimination by the complement system, while membrane-bound PRRs, like TLRs, activate signaling pathways that induce antimicrobial effector mechanisms and inflammation (12). Intercellular as well as intracellular cross-talk between TLRs and complement is known. Binding of the complement-derived anaphylatoxins C5a and C3a to their receptors—C5aR and C3aR, respectively—modulates TLR4, TLR2/6, and TLR9 signaling (14). Moreover, pentraxin 3 (PTX3), a PRR belonging to the pentraxin family, is produced by a variety of cells and tissues in response to TLR signaling and modulates complement activation through interaction with C1q and factor H (15). At the intracellular level, the complement receptor-3 (CR3), an integrin, can be transactivated by TLR2 via an inside-out signaling pathway which is distinct from the myeloid differentiation primary response protein 88 (MyD88)-dependent pro-inflammatory signaling pathway. Conversely, CR3 can initiate TLR2 and TLR4 by promoting the recruitment of the adapter molecule MyD88-adapter-like (Mal) (16). Cross-communication between TLRs and complement may serve to avoid misinterpretation of signals from non-dangerous non-self and help in the fine-tuning of the subsequent immune response to a particular microbe (17).

The missing self recognition strategy is based on molecular markers expressed on healthy cells: if these markers are missing, the cell is targeted for destruction. The major histocompatibility complex I (MHCI) proteins, constitutively expressed on all nucleated cells but often down-regulated as a result of viral infection, serve as self markers and ligands for inhibitory receptors which block the lytic activity of NK cells. Conversely, recognition of altered self is based on markers expressed only by abnormal or damaged cells, which thus are flagged for elimination (12).

## Toll-like receptors

### Discovery

The first TLR was cloned and characterized in 1997 by Ruslan Medzhitov and co-workers: under the hypothesis that the expression of co-stimulatory molecules and cytokines by antigen-presenting cells was induced by a non-clonal component of immunity, human TLR4 was discovered. It was also found that this molecule could induce activation of the nuclear factor κB (NFκB) pathway and expression of the co-stimulatory molecule B7.1, which is necessary for activation of naive T cells (18). TLRs have previously been considered solely dedicated to host defense, but evidence is emerging that these receptors may also be involved in central nervous system development and maintenance (19,20). Advances made recently in the understanding of Toll-like receptor biology in host defense and disease have been reviewed by Kawai and Akira (21).

### Evolutionary perspectives

TLR4 is the most ancient TLR, dating its origin before vertebrate life (22). Today's mammalian TLR family can be subdivided into two groups, which are the result of a gene duplication event prior to the divergence of invertebrates and vertebrates: the TLR1 family, consisting of TLRs 1, 2, 6, and 10, and a second group, comprised of all other TLRs. Orthologs of *TLR1*, *TLR6*, and *TLR10* seem to be present exclusively in mammals (23). *TLR10* separated from the *TLR1/6* precursor about 300 million years ago, roughly at the time of divergence from birds, while *TLR1* and *TLR6* appeared approximately 170 million years later (22). Following duplication, gene conversion events between *TLR1* and *TLR6*, limiting the divergence between these two genes, have been suggested (24). In mice and humans, remnants of a second, disrupted TLR2-like gene is located in tandem with the functional version of *TLR2*. This indicates the occurrence of a gene duplication after which one of the gene copies has developed into a pseudogene. Since there are two TLR2 genes in chicken, the duplication event probably occurred before the divergence of mammals and birds (25).

**Figure 2.**
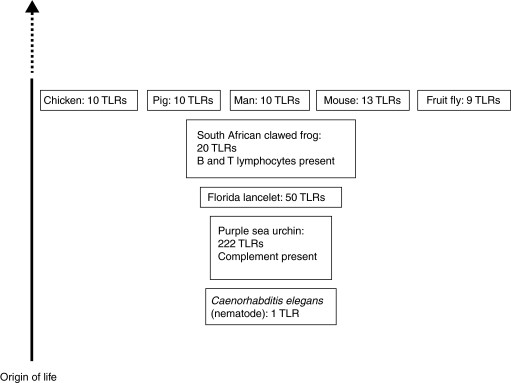
Schematic and simplified overview of the development of the immune defense. Numbers of Toll-like receptors vary between species and tend to decrease during the course of evolution. Proteins belonging to the complement system are present in the purple sea urchin. The South African clawed frog possesses an adaptive immune defense based on B and T lymphocytes.

The nematode *Caenorhabditis elegans* possesses a single TLR (Figure 2) (26). On the other hand, the genome of the purple sea urchin encodes 222 TLRs (27), demonstrating that in the absence of adaptive immunity a large repertoire of PRRs may provide a survival advantage in a pathogen-rich environment (28). The genome of the Florida lancelet is thought to harbor roughly 50 TLR genes (29), while at least 20 are known in the South African clawed frog (30).

Seventeen different TLRs are known in bony fish (7). The orthologs of TLR6 and TLR10 in mammals are absent, while TLR14—which has been found only in aquatic animals, suggesting an aquatic pathogen (31)—and TLRs 18–23 have been identified (7). Some piscine TLRs appear to have a different ligand repertoire and functional role compared to their mammalian counterparts: piscine TLR3 has been shown to respond to viral as well as bacterial MAMPs, while TLR4 may be acting as an inhibitor of the MyD88-dependent signaling pathway in zebrafish (7). TLRs also exhibit a different diversity pattern in fish compared to mammals: in rainbow trout, a soluble TLR5—possibly amplifying flagellin-induced signaling (32)—is present together with the membrane-bound version, while two TLR4 genes and two TLR8 genes have been found in zebrafish, and two splicing isoforms of *TLR9* are present in gilthead seabream and large yellow croaker (7). In comparison, soluble versions of TLR2 (33) and TLR4 (34), both with negative regulatory function towards their respective membrane-bound versions, are known in mammals.

In the chicken genome, ten TLR genes have been identified (Figure 2), one of which–C*hTLR15*—is specific for chicken (25). Ten functional TLRs are known in humans and pigs (35). In mice, TLRs 11–13 have been identified (36), but mouse *TLR10* is non-functional, due to a retroviral insertion (37). *TLR11* is present as a pseudogene in humans (38).

### Controlling the double-edged sword

Due to the potent nature of TLR signaling, TLR activation is a double-edged sword, being essential for provoking the innate immune response and enhancing the adaptive immune response, but simultaneously opening a door for pathogenesis. Negative regulation of TLR signaling is achieved at multiple levels through a large number of molecules (21). During acute bacterial infection, soluble TLRs can function as decoy receptors, preventing over-activation of the membrane-bound versions. Once ligand-receptor interaction has occurred, intracellular regulators further control TLR signaling (39). Furthermore, TLR signaling is also controlled by membrane-associated regulators, e.g. single immunoglobulin interleukin-1 receptor-related molecule (SIGIRR) (39), through miRNAs (40), by degradation of TLRs and production of anti-inflammatory cytokines as well as through apoptosis (39).

In addition to this, codon usage offers an alternative, more indirect mechanism for regulation. It has been shown that in most human TLR genes, the codon usage pattern deviates from the general one in humans, dictating a low level of protein expression (41). We have recently reported a similar difference between the general codon usage pattern in pigs and that in *TLR1*, *TLR2*, and *TLR6* (42), the alternative codon usage pattern also extending to *TLR10* (unpublished data). It is likely that this specific TLR codon usage pattern—mirroring the conditions in humans—results in protein expression at a low level. Moreover, at least in piscine TLRs, the leucine-rich repeats (LRRs) may have a role not only in ligand recognition but also in the limitation of TLR activation, since a mutant zebrafish TLR3 construct, lacking LRRs and most of the endosomal domain, showed 100 times higher reporter gene expression compared to the wild-type TLR3 construct (43).

### Comparisons to Drosophila Toll and plant TIR

Seen from an evolutionary point of view and based on the detailed structure of the extracellular domain, TLRs can be divided into vertebrate-like TLRs (V-TLRs) and protostome-like TLRs (P-TLRs) (27). Applying this approach, the *Drosophila* Toll family (Figure 2) includes eight P-TLRs (Toll included) and one V-TLR (Toll-9) (29). Phylogenetic analysis of the Toll/interleukin-1 receptor (TIR) domains in the *Drosophila* Toll family reveals that these—Toll-9 being an exception—are more closely related to one another than to TLRs in mammals. Thus, it is likely that the two groups of receptors—Toll and the *Drosophila* Toll-related receptors except Toll-9 on the one hand, and mammalian TLRs on the other—have evolved independently while carrying out different main functions (44). The difference is further emphasized by the fact that the physical interaction between mammalian TLRs and MAMPs (21) occurs in a completely different manner compared to the binding of the endogenous ligand Spätzle by Toll (45).

Flagellin-sensitive 2 (FLS2) in *Arabidopsis thaliana*, thale cress, is a transmembrane protein involved—like TLR5—in the recognition of flagellin. FLS2 is equipped with an extracellular LRR domain, but the low degree of sequence similarity between FLS2 and human TLR5 suggests that these proteins arose independently. Resistance (R) proteins containing LRR domains fused to TIR domains are present in plants, and R proteins resembling either CD14, a co-receptor in the TLR4 receptor complex, or TLRs are also known (46). In contrast to animal TIRs, plant TIR domains may interact directly with pathogen effectors, while a role in signaling for plant TIR domains remains to be established. However, some TIR domain-containing R proteins are found in the nucleus and may have roles as transcription factors. Again, these differences in the use of the TIR domain may imply convergent (47) or parallel (28) evolution rather than conservation from an early ancestor of both animals and plants.

## Mannan-binding lectin

### MBL: an initiator of the complement cascade

The role of the complement cascade in the human immune defense has recently been reviewed by Zipfel (10). MBL and ficolins initiate the lectin activation pathway of complement in co-operation with MBL-associated serine proteases (MASPs) (48). The lectin pathway is the most recently described of the main complement activation pathways (49) but possibly the most ancient: a glucose-specific lectin which interacts with MASPs has been purified from the sea pineapple (50), and an ortholog of the mammalian MBLs is present in lamprey (51). Ficolin-3 has been found to be the most potent of the lectin pathway initiators in humans, followed by ficolin-2 and MBL (52). MBL is similar in structure to C1q, a subunit of the classical pathway C1 complex, and mammalian MBL—but not MBL in chicken and fish—is thought to have the same ability as C1q to stimulate phagocytosis through the C1qRp receptor. The function of MBL/MASPs is equivalent to that of the C1 complex: C4 and C2 are cleaved, and the classical/lectin pathway C3 and C5 convertases—C4b2a and C4b2aC3b, respectively—are formed (48). Recently, a novel MBL/ficolin-associated protein, representing a short splice variant of the MASP1 gene and denoted MAP-1, has been detected in humans. This protein is expressed in myocardial and skeletal muscle and thought to inhibit the complement system by preventing the cleavage of C4 (53).

### The MBL molecule

MBL belongs to the protein family of collagenous lectins, which includes multifunctional proteins with defensive and housekeeping roles (48). The functions of collagenous lectins and TLRs are known to intersect: MBL has been found to inhibit mouse macrophage tumor necrosis factor α (TNFα) production—which is a consequence of TLR signaling leading to activator protein 1 (AP-1) activation (54)—by shielding *Blastomyces dermatitidis* epitopes (55), while *in-vitro* experiments have shown a dampening effect of surfactant protein A (SP-A) on pulmonary inflammation caused by zymosan, probably through the binding of SP-A to TLR2 (56). In most mammals, two forms of MBL—MBL-A and MBL-C—are present. These are encoded by separate genes, denoted *MBL1* and *MBL2*. Duplication of the MBL gene probably occurred after the divergence of birds and mammals, since only one MBL gene is present in chicken. In humans and chimpanzee, *MBL1* is a pseudogene (48).

The basic subunit of mammalian MBL is a trimer composed of three identical monomers, each consisting of four distinct domains: a cysteine-rich N-terminal domain, a collagen-like domain, a neck region, and a C-terminal carbohydrate recognition domain. The collagen-like domain is composed of numerous G-X-Y motifs (X and Y representing any amino acid), typical of a helix structure. If a glycine in a G-X-Y motif is replaced by a more bulky amino acid, the formation of the trimer triple helix is disturbed. This, in turn, impairs the functionality of the molecule, since this is dependent on oligomerization of the basic trimers (57). The binding of the functional MBL molecule to the pathogen is, in turn, dependent on MBLs' steric specificity and the spatial organization of the carbohydrate ligands on the pathogenic surface; conversely, self tissue is spared by these requirements (50). In serum, MBL exists in complex with MASPs 1–3 and small MBL-associated protein (sMAP), the latter representing a short splice variant of the MASP2 gene and playing a regulatory role in the lectin pathway. When MBL binds to a cell surface displaying a fitting carbohydrate pattern, the proenzyme forms of MASPs are cleaved and become proteolytically active (58).

### Differences between species

The abundance, forms, and functions of the collagenous lectins vary considerably between species. In humans, one MBL and three ficolins are expressed, while there are two ficolins and two MBLs in most other species. Furthermore, in MBL and ficolins, the recognition domains are aggregated at one pole, which creates their sertiform (‘bunch of tulips’) formation. This facilitates multivalent binding to monosaccharides on microbial surfaces. In contrast, in the bovine-specific conglutinin (CG) and collectin-46 (CL-46), the recognition domains are distributed at diametric poles, leading to a cruciform which facilitates agglutination of targets; however, these cruciform molecules have not been shown to activate complement. Moreover, unlike other collagenous lectin trimers, those of bovine-specific collectin-43 (CL-43) and human collectin liver 1 (CL-L1) do not assemble into higher-order oligomers. Differences like these may reflect co-evolution with relevant pathogens (48).

## *TLRs* and *MBL*: finding Mr Darwin—and not finding him

### Toll-like receptors

The weaknesses of some widely used statistical methods aiming to identify positive (Darwinian) selection by comparisons of numbers of non-synonymous and synonymous single nucleotide polymorphisms (SNPs) have been pointed out by Hughes and co-workers (59). If a protein is evolving under positive selection, then a pattern of dN>dS (i.e. the proportionate number of non-synonymous differences, dN, exceeding the proportionate number of synonymous differences, dS) is expected, as has been found regarding the peptide-binding region (PBR) of the MHC molecules. MHC proteins present peptide antigens to antigen-specific T cells. Since MHC molecules encoded by different alleles present different antigens, the advantage of a heterozygote in terms of increased peptide-presentation capacity maintains MHC polymorphism. Thus, from a biological point of view, positive selection repeatedly favoring amino acid changes in the PBR is reasonable. However, this pattern may not be characteristic for positive selection in general. The methods based on comparisons of non-synonymous and synonymous SNPs often fail to rule out alternative hypotheses, in particular the relaxation of purifying selection and the effects of population bottle-necks (59). Furthermore, the dN/dS statistic, originally developed for quantification of selective pressure in divergent species, has also been scrutinized and challenged. Even though it is traditionally used to quantify selection pressure within populations, it has been shown to be inadequate when applied in this manner or for closely related taxa (60,61).

It is known that many pathogens, among them *Yersinia pestis*, express lipid A structures which do not fully stimulate TLR4 (62), demonstrating that at least some MAMPs do evolve in response to selective pressure. Moreover, there is accumulating evidence of a species-specific component in TLR function, which, in turn, might reflect co-evolution with relevant micro-organisms (63). Bovine TLR2/TLR1 (63) and chicken TLR2-type2/TLR1-type2 (64) both display broader ligand specificities than the corresponding receptor complexes in most mammals. *TLR2* in ruminants seems to be subject to a lower degree of selection compared to *TLR2* in other mammals (63), and polymorphism in bovine *TLR2* is known to occur mainly between breeds, the genotype in a particular breed probably reflecting the microflora at hand (65). Furthermore, certain wild-derived inbred strains of mice differ in TLR3 genotype from classical laboratory strains (66). Our own findings, showing that wild boars and domestic pigs display different polymorphic patterns in the TLR1, TLR2, TLR6 (42), and TLR10 (unpublished data) genes, parallel these observations and further emphasize that different microbial environments are involved in shaping the differences in genotypes observed between populations/strains. To summarize, positive selection acting on the ligand-binding domains of TLRs appears reasonable from a biological point of view. Indeed, the pattern observed in an analysis of 22 mammalian *TLR2* sequences parallels that of MHC: amino acids responsible for the binding of MAMPs evolve under positive selection, while regions important for heterodimerization—especially the TIR domain—are subject to purifying selection (63). Moreover, a similar pattern has been reported concerning bovine *TLR4* (67). Furthermore, biased distributions of non-synonymous SNPs, compatible with different modes of selection acting on different parts of the genes, have been reported regarding porcine TLR genes (68,69). Also, in bony fish, certain sites in the LRR domains of TLR9 seem to evolve under positive selection (70).

Barreiro and co-workers (71) reported evidence for population-specific events of positive selection on the TLR10-1-6 gene cluster, strong purifying selection acting on the intracellular TLRs, and more relaxed selective constraint on cell surface TLRs in humans. Taken as a set, human TLRs have evolved under purifying selection according to this study (71). In most TLRs, Wlasiuk and co-workers detected positive selection between humans and chimpanzees, as opposed to purifying selection within species (72). Also, a comparison between human and rhesus monkey orthologous gene pairs revealed significantly lower dN—and thus greater functional constraint—on the TIR domain compared to LRRs in TLR4, TLR5, TLR7, TLR8, and TLR9, but no positive selection was reported (23). Bovine *TLR10* seems to evolve under strong purifying selection, indicating an important function for the corresponding receptor. This is interesting, since TLR10 is the only member of the TLR family for which no ligand is known. It remains to be defined what kind of functional constraint might be important enough to justify strong purifying selection (73). In contrast, relaxed selective constraint, indicating some degree of redundancy, has been suggested for human *TLR10*: in the large-scale screening project undertaken by Barreiro and co-workers, *TLR10* was found to be by far the most variable of the human TLR genes (71). Due to markedly different physiologies, the pathogenic load and the balance between commensals and pathogens are likely to differ between ruminants and non-ruminant species. Thus, species-specific function and co-evolution with relevant micro-organisms might explain why bovine and human *TLR10* seem to be subject to different modes of selection. Our own findings regarding polymorphic patterns in the TLR1, TLR2, TLR6 (42), and TLR10 (unpublished data) genes in pigs point in the direction of purifying selection: visualizations of the amino acid positions corresponding to detected non-synonymous SNPs, using the crystal structure determined for the human TLR1-TLR2-lipopeptide complex (74) combined with protein sequence alignments, indicated that the majority of the variable positions are distinct from the ligand-binding site and the dimerization interface.

It has been argued that positive selection detectable as a pattern of dN>dS, indicating repeated amino acid changes at a limited set of codons, is likely to be very rare. Comparisons of dN and dS would not detect single non-synonymous changes at single codons, whole or partial gene deletion, or changes in the patterns of gene expression; however, such punctual events are known to produce adaptive genotypes and likely to be more common (59). Moreover, it has been pointed out that the highly mutable CpG positions are much more common in replacement sites in codons than in introns, rendering the fundamental assumption of equal mutation rates in different parts of the genome unjustifiable. Thus, the intensity of purifying selection on coding sequences may be greater than previously inferred (75). In agreement with this, Roach and co-workers found no support for positive selection in the vertebrate TLR phylogeny through evaluation of synonymous/non-synonymous substitution ratios (36). In conclusion, despite their potential as a parallel to the MHC proteins as far as patterns of selection are concerned, a conservative approach regarding the extent to which positive selection is acting on and has been detected in TLRs still seems appropriate.

### Mannan-binding lectin

In the coding sequence of the human MBL2 gene, three non-synonymous SNPs, all with decreasing effects on MBL concentration, have been identified. Two of these SNPs implicate the replacement of a glycine residue in a G-X-Y motif with an aspartic acid (the B allele) and a glutamic acid (the C allele), respectively. Furthermore, three promoter polymorphisms affecting MBL serum concentrations are known. Low-producing MBL alleles are present at varying frequencies in different human populations (76). MBL deficiency has been found to increase susceptibility to many infectious diseases (77), but also, in contrast, to increase resistance against leishmaniasis (78) and tuberculosis (TB) (79). However, in the case of TB, the existing view has been challenged. In a recent meta-analysis carried out by Denholm and co-workers (80), no significant association was found between MBL2 genotype and pulmonary TB infection. However, the majority of the analyses did not report MBL2 haplotypes inclusive of promoter polymorphisms. MBL serum concentrations were consistently elevated during TB infection, but this increase was also of a degree consistent with the acute-phase reaction. If indeed MBL deficiency does not protect against TB, it is challenging to propose a different disease that may have promoted the high frequency of low-producing MBL alleles observed in some human populations (80). It is believed that during evolution, the human MBL1 gene has been turned off by the same molecular mechanisms causing the variant MBL2 alleles, i. e. mainly through mutations affecting the glycines in the G-X-Y motifs in the collagen-like domain. This is consistent with the hypothesis that low MBL levels represent a selective advantage (81). In the coding sequence of porcine *MBL1*, a non-synonymous SNP, implicating the replacement of the glycine in G-X-Y motif 16 with a cysteine, has been detected. This SNP is present at different frequencies in various domestic pig populations (82) and is assumed to affect MBL-A concentration in serum (83). The existence of this SNP in porcine *MBL1* may be an indication of an on-going evolutionary process favoring low-producing MBL alleles, mirroring what might be the case in humans. However, the hypothesis of selection for low-producing MBL alleles in human populations is not undisputed: balancing selection, implying an advantage for heterozygotes (84), as well as neutral evolution and redundancy (85) has been suggested. The study by Verdu and co-workers (85) is based on a large-scale sequencing project, and data have been analyzed through several methods. However, bearing in mind the importance of biological reasoning for conclusions about modes of evolution, it seems wise not to dismiss the study by Seyfarth and co-workers (81), since it is based on comparisons between functional and non-functional MBL1 genes in humans and other primates.

### Concluding remarks

There seems to be a number of genes encoding defense and/or immunity proteins which have evolved rapidly since the divergence of humans and chimpanzees (86). However, Enard and co-workers have recently shown (87) that positive selection acting simultaneously on the same genes in humans and other primates is not uncommon and not restricted to a particular function, e.g. immune capacity. On the other hand, independent selective sweeps in human, chimpanzee, and orangutan were inferred for the TLR6-1-10 gene cluster (87). Also, immune-related genes are over-represented when recently occurring selective events in human populations are considered. The invention of farming—which led to a massive increase of human population sizes, possibly facilitating the spread of certain infectious agents—and exposure to new zoonoses following the domestication of animals may have posed strong challenges on the human immune system (86).

Recently, the TIR protein family has been pointed out as an example where the existence of a limited number of protein domains with relevant functions has constrained evolution, resulting in the emergence of proteins with identical architectures but lacking orthologous relationship and performing different functions in different lineages, the *Drosophila* Toll proteins and the vertebrate TLRs being examples. There are several examples of PRR domain architectures which have emerged multiple times, suggesting that parallel evolution may be a common phenomenon in innate immunity evolution (28). Concerning to what extent positive selection is acting on TLR genes, evidence is somewhat contradictive. This might, at least in part, be explained by differences between species and by which SNPs are present in the particular animal materials used to produce data sets. However, the biological function of TLRs does not exclude the possibility that they are parallel to the MHC molecules as far as patterns of selection are concerned. In general, the commonly accepted arms race model of interaction between pathogens and their hosts needs to be complemented by a conception of adaptive evolution as consisting of a series of leaps, as it seems obvious—from a theoretical point of view (59) and as a result of laboratory-based research (72)—that adaptive evolution often is episodic.

## References

[1] Palm NW, Medzhitov R (2009). Pattern recognition receptors and control of adaptive immunity. Immunol Rev.

[2] Pangburn MK, Ferreira VP, Cortes C (2008). Discrimination between host and pathogens by the complement system. Vaccine.

[3] Crocker PR, Paulson JC, Varki A (2007). Siglecs and their roles in the immune system. Nat Rev Immunol.

[4] vanBeek EM, Cochrane F, Barclay AN, van den Berg TK (2005). Signal regulatory proteins in the immune system. J Immunol.

[5] Venkatesh B (2003). Evolution and diversity of fish genomes. CurrOpin Genet Dev.

[6] Magnadóttir B (2006). Innate immunity of fish (overview). Fish Shellfish Immunol.

[7] Rebl A, Goldammer T, Seyfert HM (2010). Toll-like receptor signaling in bony fish. Vet ImmunolImmunopathol.

[8] Nikolakopoulou K, Zarkadis IK (2006). Molecular cloning and characterisation of two homologues of Mannose-Binding Lectin in rainbow trout. Fish Shellfish Immunol.

[9] Zarkadis IK, Mastellos D, Lambris JD (2001). Phylogenetic aspects of the complement system. Dev Comp Immunol.

[10] Zipfel PF (2009). Complement and immune defense: from innate immunity to human diseases. ImmunolLett.

[11] Kabelitz D, Medzhitov R (2007). Innate immunity-cross-talk with adaptive immunity through pattern recognition receptors and cytokines. CurrOpinImmunol.

[12] Medzhitov R, Janeway CA (2002). Decoding the patterns of self and nonself by the innate immune system. Science.

[13] Medzhitov R (2001). Toll-like receptors and innate immunity. Nat Rev Immunol.

[14] Zhang X, Kimura Y, Fang C, Zhou L, Sfyroera G, Lambris JD (2007). Regulation of Toll-like receptor-mediated inflammatory response by complement in vivo. Blood.

[15] Garlanda C, Maina V, Cotena A, Moallia F (2009). The soluble pattern recognition receptor pentraxin-3 in innate immunity, inflammation and fertility. J ReprodImmunol.

[16] Hajishengallis G, Lambris JD (2010). Crosstalk pathways between Toll-like receptors and the complement system. Trends Immunol.

[17] Friec GL, Kemper C (2009). Complement: coming full circle. Arch ImmunolTherExp (Warsz).

[18] Medzhitov R, Preston-Hurlburt P, Janeway CA (1997). A human homologue of the Drosophila Toll protein signals activation of adaptive immunity. Nature.

[19] Larsen PH, Holm TH, Owens T (2007). Toll-like receptors in brain development and homeostasis. SciSTKE.

[20] Ma Y, Li J, Chiu I, Wang Y, Sloane JA, Lü J (2006). Toll-like receptor 8 functions as a negative regulator of neurite outgrowth and inducer of neuronal apoptosis. J Cell Biol.

[21] Kawai T, Akira S (2010). The role of pattern-recognition receptors in innate immunity: update on Toll-like receptors. Nat Immunol.

[22] Beutler B, Rehli M (2002). Evolution of the TIR, Tolls and TLRs: functional inferences from computational biology. Curr Top MicrobiolImmunol.

[23] Hughes AL, Piontkivska H (2008). Functional diversification of the toll-like receptor gene family. Immunogenetics.

[24] Kruithof EKO, Satta N, Liu JW, Dunoyer-Geindre S, Fish RJ (2007). Gene conversion limits divergence of mammalian TLR1 and TLR6. BMC Evol Biol.

[25] Boyd A, Philbin VJ, Smith AL (2007). Conserved and distinct aspects of the avian Toll-like receptor (TLR) system: implications for transmission and control of bird-borne zoonoses. BiochemSoc Trans.

[26] Tenor JL, Aballay A (2008). A conserved Toll-like receptor is required for Caenorhabditis elegans innate immunity. EMBO Rep.

[27] Hibino T, Loza-Coll M, Messier C, Majeske AJ, Cohen AH, Terwilliger DP (2006). The immune gene repertoire encoded in the purple sea urchin genome. Dev Biol.

[28] Zhang Q, Zmasek CM, Godzik A (2010). Domain architecture evolution of pattern-recognition receptors. Immunogenetics.

[29] Huang S, Yuan S, Guo L, Yu Y, Li J, Wu T (2008). Genomic analysis of the immune gene repertoire of amphioxus reveals extraordinary innate complexity and diversity. Genome Res.

[30] Ishii A, Kawasaki M, Matsumoto M, Tochinai S, Seya T (2007). Phylogenetic and expression analysis of amphibian Xenopus Toll-like receptors. Immunogenetics.

[31] Ishii A, Matsuo A, Sawa H, Tsujita T, Shida K, Matsumoto M (2007). Lamprey TLRs with properties distinct from those of the variable lymphocyte receptors. J Immunol.

[32] Tsujita T, Tsukada H, Nakao M, Oshiumi H, Matsumoto M, Seya T (2004). Sensing bacterial flagellin by membrane and soluble orthologs of Toll-like receptor 5 in rainbow trout (Onchorhynchusmikiss). J Biol Chem.

[33] LeBouder E, Rey-Nores JE, Rushmere NK, Grigorov M, Lawn SD, Affolter M (2003). Soluble forms of Toll-like receptor (TLR)2 capable of modulating TLR2 signaling are present in human plasma and breast milk. J Immunol.

[34] Iwami KI, Matsuguchi T, Masuda A, Kikuchi T, Musikacharoen T, Yoshikai Y (2000). Cutting edge: naturally occurring soluble form of mouse Toll-like receptor 4 inhibits lipopolysaccharide signaling. J Immunol.

[35] Uenishi H, Shinkai H (2008). Porcine Toll-like receptors: the front line of pathogen monitoring and possible implications for disease resistance. Dev Comp Immunol.

[36] Roach JC, Glusman G, Rowen L, Kaur A, Purcell MK, Smith KD (2005). The evolution of vertebrate Toll-like receptors. ProcNatlAcadSci U S A.

[37] Hasan U, Chaffois C, Gaillard C, Saulnier V, Merck E, Tancredi S (2005). Human TLR10 is a functional receptor, expressed by B cells and plasmacytoid dendritic cells, which activates gene transcription through MyD88. J Immunol.

[38] Takeda K, Akira S (2005). Toll-like receptors in innate immunity. IntImmunol.

[39] Liew FY, Xu D, Brint EK, O'Neill LA (2005). Negative regulation of toll-like receptor-mediated immune responses. Nat Rev Immunol.

[40] Hennessy EJ, O'Neill LAJ Toll-like receptors: very clever molecules. http://www.abcam.com/index.html?pageconfigresource&rid12189&pid10629.

[41] Zhong F, Cao W, Chan E, Tay PN, Cahya FF, Zhang H (2005). Deviation from major codons in the Toll-like receptor genes is associated with low Toll-like receptor expression. Immunology.

[42] Bergman IM, Rosengren JK, Edman K, Edfors I (2010). European wild boars and domestic pigs display different polymorphic patterns in the Toll-like receptor (TLR) 1, TLR2, and TLR6 genes. Immunogenetics.

[43] Phelan PE, Mellon MT, Kim CH (2005). Functional characterization of full-length TLR3, IRAK-4, and TRAF6 in zebrafish (Daniorerio). MolImmunol.

[44] Bilak H, Tauszig-Delamasure S, Imler JL (2003). Toll and Toll-like receptors in Drosophila. BiochemSoc Trans.

[45] Gangloff M, Murali A, Xiong J, Arnot CJ, Weber AN, Sandercock AM (2008). Structural insight into the mechanism of activation of the Toll receptor by the dimeric ligand Spätzle. J Biol Chem.

[46] Nürnberger T, Brunner F, Kemmerling B, Piater L (2004). Innate immunity in plants and animals: striking similarities and obvious differences. Immunol Rev.

[47] Burch-Smith TM, Dinesh-Kumar SP (2007). The functions of plant TIR domains. SciSTKE.

[48] Lillie BN, Brooks AS, Keirstead ND, Hayes MA (2005). Comparative genetics and innate immune functions of collagenous lectins in animals. Vet ImmunolImmunopathol.

[49] Ikeda K, Sannoh T, Kawasaki N, Kawasaki T, Yamashina I (1987). Serum lectin with known structure activates complement through the classical pathway. J Biol Chem.

[50] Fujita T, Matsushita M, Endo Y (2004). The lectin-complement pathway-its role in innate immunity and evolution. Immunol Rev.

[51] Takahashi M, Iwaki D, Matsushita A, Nakata M, Matsushita M, Endo Y (2006). Cloning and characterization of mannose-binding lectin from lamprey (Agnathans). J Immunol.

[52] Hummelshoj T, Fog LM, Madsen HO, Sim RB, Garred P (2008). Comparative study of the human ficolins reveals unique features of Ficolin-3 (Hakata antigen). MolImmunol.

[53] Skjoedt MO, Hummelshoj T, Palarasah Y, Honore C, Koch C, Skjodt K (2010). A novel mannose-binding lectin/ficolin-associated protein is highly expressed in heart and skeletal muscle tissues and inhibits complement activation. J Biol Chem.

[54] Brikos C, O'Neill AJO, Bauer S, Hartmann G (2008). Signalling of Toll-like receptors. Toll-like receptors (TLRs) and innate immunity.

[55] Koneti A, Linke MJ, Brummer E, Stevens DA (2008). Evasion of innate immune responses: evidence for mannose binding lectin inhibition of tumor necrosis factor alpha production by macrophages in response to Blastomyces dermatitidis. Infect Immun.

[56] Sato M, Sano H, Iwaki D, Kudo K, Konishi M, Takahashi H (2003). Direct binding of Toll-like receptor 2 to zymosan, and zymosan-induced NF-kappa B activation and TNF-alpha secretion are down-regulated by lung collectin surfactant protein A. J Immunol.

[57] Garred P, Larsen F, Seyfarth J, Fujita R, Madsen HO (2006). Mannose-binding lectin and its genetic variants. Genes Immun.

[58] Iwaki D, Kanno K, Takahashi M, Endo Y, Lynch NJ, Schwaeble WJ (2006). Small mannose-binding lectin-associated protein plays a regulatory role in the lectin complement pathway. J Immunol.

[59] Hughes AL (2007). Looking for Darwin in all the wrong places: the misguided quest for positive selection at the nucleotide sequence level. Heredity.

[60] Kryazhimskiy S, Plotkin JB (2008). The population genetics of dN/dS. PLoS Genet.

[61] Wolf JB, Künstner A, Nam K, Jakobsson M, Ellegren H (2009). Nonlinear dynamics of nonsynonymous (dN) and synonymous (dS) substitution rates affects inference of selection. Genome BiolEvol.

[62] Portnoy DA (2005). Manipulation of innate immunity by bacterial pathogens. CurrOpinImmunol.

[63] Werling D, Jann OC, Offord V, Glass EJ, Coffey TJ (2009). Variation matters: TLR structure and species-specific pathogen recognition. Trends Immunol.

[64] Higuchi M, Matsuo A, Shingai M, Shida K, Ishii A, Funami K (2008). Combinational recognition of bacterial lipoproteins and peptidoglycan by chicken Toll-like receptor 2 subfamily. Dev Comp Immunol.

[65] Jann OC, Werling D, Chang JS, Haig D, Glass EJ (2008). Molecular evolution of bovine Toll-like receptor 2 suggests substitutions of functional relevance. BMC Evol Biol.

[66] Stephan K, Smirnova I, Jacque B, Poltorak A (2007). Genetic analysis of the innate immune responses in wild-derived inbred strains of mice. Eur J Immunol.

[67] White SN, Taylor KH, Abbey CA, Gill CA, Womack JE (2003). Haplotype variation in bovine Toll-like receptor 4 and computational prediction of a positively selected ligand-binding domain. ProcNatlAcadSci U S A.

[68] Shinkai H, Tanaka M, Morozumi T, Eguchi-Ogawa T, Okumura N, Muneta Y (2006). Biased distribution of single nucleotide polymorphisms (SNPs) in porcine Toll-like receptor 1 (TLR1), TLR2, TLR4, TLR5, and TLR6 genes. Immunogenetics.

[69] Morozumi T, Uenishi H (2009). Polymorphism distribution and structural conservation in RNA-sensing Toll-like receptors 3, 7, and 8 in pigs. BiochimBiophysActa.

[70] Chen JS, Wang TY, Tzeng TD, Wang CY, Wang D (2008). Evidence for positive selection in the TLR9 gene of teleosts. Fish Shellfish Immunol.

[71] Barreiro LB, Ben-Ali M, Quach H, Laval G, Patin E, Pickrell JK (2009). Evolutionary dynamics of human Toll-like receptors and their different contributions to host defense. PLoS Genet.

[72] Wlasiuk G, Nachman MW (2010). Adaptation and constraint at Toll-like receptors in primates. MolBiolEvol.

[73] Seabury CM, Seabury PM, Decker JE, Schnabel RD, Taylor JF, Womack JE (2010). Diversity and evolution of 11 innate immune genes in Bostaurustaurus and Bostaurusindicus cattle. ProcNatlAcadSci U S A.

[74] Jin MS, Kim SE, Heo JY, Lee ME, Kim HM, Paik SG (2007). Crystal structure of the TLR1-TLR2 heterodimer induced by binding of a tri-acylated lipopeptide. Cell.

[75] Subramanian S, Kumar S (2006). Higher intensity of purifying selection on >90% of the human genes revealed by the intrinsic replacement mutation rates. MolBiolEvol.

[76] Garred P, Honoré C, Ma YJ, Munthe-Fog L, Hummelshøj T (2009). MBL2, FCN1, FCN2 and FCN3-the genes behind the initiation of the lectin pathway of complement. MolImmunol.

[77] Turner MW (2003). The role of mannose-binding lectin in health and disease. MolImmunol.

[78] Santos IK, Costa CH, Krieger H, Feitosa MF, Zurakowski D, Fardin B (2001). Mannan-binding lectin enhances susceptibility to visceral leishmaniasis. Infect Immun.

[79] Garred P, Richter C, Andersen AB, Madsen HO, Mtoni I, Svejgaard A (1997). Mannan-binding lectin in the sub-Saharan HIV and tuberculosis epidemics. Scand J Immunol.

[80] Denholm JT, McBryde ES, Eisen DP (2010). Mannose-binding lectin and susceptibility to tuberculosis: a meta-analysis. ClinExpImmunol.

[81] Seyfarth J,Garred P, Madsen HO (2005). The ‘involution’ of mannose-binding lectin. Hum Mol Genet.

[82] Lillie BN, Keirstead ND, Squires EJ, Hayes MA (2006). Single-hnucleotide polymorphisms in porcine mannan-binding lectin A. Immunogenetics.

[83] Juul-Madsen HR, Krogh-Meibom T, Henryon M, Palaniyar N, Heegaard PM, Purup S (2007). Identification and characterization of porcine mannan-binding lectin A (pMBL-A), and determination of serum concentration heritability. Immunogenetics.

[84] Bernig T, Taylor JG, Foster CB, Staats B, Yeager M, Chanock SJ (2004). Sequence analysis of the mannose-binding lectin (MBL2) gene reveals a high degree of heterozygosity with evidence of selection. Genes Immun.

[85] Verdu P, Barreiro LB, Patin E, Gessain A, Cassar O, Kidd JR (2006). Evolutionary insights into the high worldwide prevalence of MBL2 deficiency alleles. Hum Mol Genet.

[86] Barreiro LB, Quintana-Murci L (2010). From evolutionary genetics to human immunology: how selection shapes host defence genes. Nat Rev Genet.

[87] Enard D, Depaulis F, Crollius HR (2010). Human and non-human primate genomes share hotspots of positive selection. PLoS Genet.

